# Passive Control of Silane Diffusion for Gradient Application of Surface Properties

**DOI:** 10.3390/mi12111360

**Published:** 2021-11-04

**Authors:** Riley L. Howard, Francesca Bernardi, Matthew Leff, Emma Abele, Nancy L. Allbritton, Daniel M. Harris

**Affiliations:** 1 Department of Applied Physical Sciences, University of North Carolina at Chapel Hill, Chapel Hill, NC 27599, USA; mleff@med.unc.edu; 2 Department of Mathematical Sciences, Worcester Polytechnic Institute, Worcester, MA 01609, USA; 3 School of Engineering, Brown University, Providence, RI 02912, USA; emma_abele@alumni.brown.edu (E.A.); daniel_harris3@brown.edu (D.M.H.); 4 Department of Bioengineering, University of Washington, Seattle, WA 98195, USA; nlallbr@uw.edu

**Keywords:** liquid lithography, silanization, Monte Carlo, gradient, wettability, micropillar, diffusion, grafting, lab-on-a-chip

## Abstract

Liquid lithography represents a robust technique for fabricating three-dimensional (3D) microstructures on a two-dimensional template. Silanization of a surface is often a key step in the liquid lithography process and is used to alter the surface energy of the substrate and, consequently, the shape of the 3D microfeatures produced. In this work, we present a passive technique that allows for the generation of silane gradients along the length of a substrate. The technique relies on a secondary diffusion chamber with a single opening, leading to a directional introduction of silane to the substrate via passive diffusion. The secondary chamber geometry influences the deposited gradient, which is shown to be well captured by Monte Carlo simulations that incorporate the passive diffusion and grafting processes. The technique ultimately allows the user to generate a range of substrate wettabilities on a single chip, enhancing throughput for organ-on-a-chip applications by mimicking the spatial variability of tissue topographies present in vivo.

## 1. Introduction

Silicon–hydrocarbon compounds, known as organosilanes, play an important role as a surface treatment for microfluidic devices in a process known as silanization [1]. Silanization typically involves the liquid or vaporous deposition of organosilanes onto a microfluidic surface whose physical properties change as a result [2]. Organosilanes are particularly useful in surface modification of solids because hydroxyl (and other hydrocarbon) groups can bind strongly and steadily with the silicon atoms within the silane, allowing solid monolayers of silane to form across an appropriate surface [3]. Monolayer coatings on microsurfaces act as attachment sites for various ligands and can functionalize surfaces for stronger ligand specificity [4]. In general, silanization is an extremely valuable technique in bioengineering as it greatly increases surface functionality and can be used to augment cell and protein adhesion [5]. Applications of organosilane coatings include binding nucleotides and DNA to a surface coated with an amine-bonded organosilane layer [6], mitigating the extreme hydrophobicity of native polydimethysiloxane [7], treating a hydroxypropyl methylcellulose hydrogel scaffold for chondrocyte differentiation [8], and treating porous chitosan scaffolds for improved degradation rates [5].

Silanization is also frequently used in liquid lithography—a 3D-microstructure manufacturing technique that relies on the interaction of two immiscible liquids and leverages their interfacial tension to form a surface of controlled curvature [9]. Liquids with different relative surface tensions create reproducible patterned features when placed within a photolithographic template. The curved surfaces formed can range from relatively flat interfaces when liquids of similar surface energies are employed, to extreme curvatures for the case of liquids with very different surface energies [10]. Liquid lithography aims to resolve one of the major roadblocks in properly mimicking in vivo biological applications with in vitro cell culture: reproducing the wide range of physical microstructures that occur in native tissue [11]. Tissue topography is challenging to replicate in vitro [12,13] as it often displays variations in microcurvature, physical properties, and topology even within a single tissue type [14,15]. Despite its effectiveness in producing 3D microstructures with both concave and convex geometries, liquid lithography presents challenges when manufacturing curved surfaces with varying shapes in the same batch. For example, liquid lithography is not yet capable of producing microraft arrays with variable microwell curvatures—which would mimic changing topography and physical properties typical of in vivo tissues. A potential way around this limitation is to employ silanization to achieve material gradients on a cell scaffold. A silane coating gradient can lead to a corresponding gradient in surface properties [16,17,18,19]. Leveraging silanization in liquid lithography can ultimately allow the user to create a microdevice with varying topographical surface properties that is more hospitable for fluid flow and cell growth [10,20,21,22].

Both active and passive strategies are commonly used to apply gradients of properties on (or in) a material. Generally, the active creation of material property gradients can be achieved in a variety of ways, including controlled fluid flow (such as in microfluidics), light patterning, and compositional control [23]. These methods are designed to facilitate tunable modifications on (or in) a material rather than relying on thermal motion and entropy to drive these changes [23]. Active methodologies have been successfully applied, for example, by creating gradients of mechanical stiffness via microfluidic addition of a crosslinker to a hydrogel [24], and by using ultraviolet light sources to crosslink photoreactive hydrogels in a gradient along a surface [25]. For the case of silanization, active methods such as ‘controlled-rate infusion’ [26] have been successfully shown to allow for the application of nearly arbitrary gradient profiles. While active methods can offer a high degree of control for gradients of certain properties, they also require specific, costly materials and equipment, as well as longer experimental run times [27].

In contrast, passive methods rely on naturally occurring physical processes (such as molecular diffusion) and only require simple protocols and little-to-no additional equipment. For instance, molecular diffusion can be leveraged to produce concentration gradients by non-uniformly introducing molecules which diffuse due to thermal motion and expand into areas of low concentration [28]. Examples include gradients of chemical crosslinking within a hydrogel [29,30] and gradients of increasing stiffness on ECM scaffolds [31]. Passive molecular diffusion of vaporous silane defines a common method to generate a wettability gradient along a substrate [32,33,34], with the equilibrium contact angle increasing with silane surface coverage. However only very few supplementary techniques have been documented to further control or predict the shape of the ultimate gradient [35]. In comparison to liquid-phase deposition, vapor-phase deposition of silane provides advantages in reducing hydrolysis and polymerization of the organosilane by reducing surface water interactions during the deposition process [36]. In addition, slower deposition timescales in vapor-phase deposition allow for better control and a higher degree of homogeneity in deposited layer thickness [36,37].

In this manuscript, we describe an experimental method to passively generate a wettability gradient suitable to influence mechanical surface properties during lithographic synthesis. We vaporize organosilane and let it passively diffuse in the interior of a small secondary rectangular chamber. The silanization process is shown to produce a coating gradient as silane deposits along a treated glass slide. The behavior of vaporized silane molecules is modeled as undergoing a diffusion-grafting process simulated using a 2D Monte Carlo method. We compare experimental measurements to predictions of this simplified mathematical model which captures the principal physics of our system and reproduces many of the qualitative trends observed in the experiment.

## 2. Materials and Methods

### 2.1. Experiments

**Figure 1:**
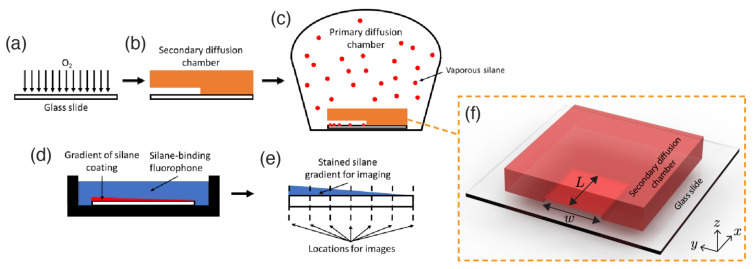
Schematic of gradient aminosilane diffusion and subsequent staining/imaging. (**a**) A clean glass slide is treated with oxygen plasma for 5 min. (**b**) A 3D-printed secondary diffusion chamber (detailed in (**f**)) is attached to the plasma-treated side of the surface. This creates one opening for the silane to penetrate into the secondary diffusion chamber. (**c**) The glass slide with secondary diffusion chamber and petri dish containing liquid-phase aminosilane are placed within the primary diffusion chamber. The primary chamber is then placed under vacuum, driving the aminosilane into the vapor phase. The chambers are incubated for a time that is insufficient for the silane to saturate the secondary diffusion chamber. (**d**) After a gradient is formed on the secondary chamber floor, the glass slide is removed from the primary diffusion chamber, detached from the secondary chamber, and incubated with an amine-specific fluorophore. Due to the monolayer nature of the silane coating, the resultant gradient is in the molecular surface coverage per unit area, and is depicted in this step as a continuous curve. (**e**) Linear transverse scans along the slide’s longitudinal axis are conducted every 5 mm. (**f**) Rendering of 3D-printed secondary diffusion chamber on glass slide.

We developed a silanization method to obtain a gradient of grafted aminosilane in a controlled manner. The aminosilane is vaporized via vacuum exposure within the overall system. The addition of a secondary, smaller chamber with only one opening, allows for directional control of the diffusion process of the vaporized silane into the vacuum space. To this end, a glass slide is attached to a secondary diffusion chamber of fixed width, height, and length. Prior to use, standard glass slides are cleaned with 70% ethanol, deionized water, and dried with compressed nitrogen. The slides are then treated with oxygen plasma for 5 min (Figure 1a). Inside a polycarbonate desiccator (08-642-7; Thermo Fisher, Fremont, CA, USA), a glass slide is placed with the plasma-treated side facing upwards. The secondary 3D-printed rectangular chamber has one side open, and is taped on top of the glass slide (Figure 1b). The chamber dimensions length × width × height are defined as L×w×h, as shown in Figure 1f. While we keep the chamber width as 30 mm, we test several chamber aspect-ratios obtained by varying its height and length. Specifically, we use chambers with three different heights (1 mm, 2 mm, 4 mm) and lengths (20 mm, 30 mm, 50 mm) to isolate the influence of the secondary chamber geometry on the silane gradient. All secondary diffusion chambers are initially rendered on Solidworks CAD software (version 2016, SolidWorks Corp., Waltham, MA, USA). The 3D printing filament used is polylactic acid (PLA), and the 3D printer used is the Prusa i3 MK3 (Prusalab; Czechia).

A volume of liquid-phase (3-aminopropyl)triethoxysilane (APTES) is deposited in a petri dish in the center of the desiccator; APTES volume is 100 µL with molar concentration 0.06 $\mathrm{mol}/m^{3}$. APTES is chosen due to an exposed amine group, allowing for an amine-specific fluorescent dye to be used to conjugate the silane and provide a quantifiable fluorescence signal. Surfaces functionalized with APTES deposition are successfully used in cell culture, modifying cell viability and the mechanical properties of scaffolds [38,39]. The chamber is placed under a 95 kPa vacuum for 5 min to vaporize the silane, then left vacuum sealed for a period of time between 2–24 h to allow for diffusion of the silane into the secondary chamber and across the surface of the glass slide (Figure 1c). Once vaporized and spread within the larger chamber, the silane concentration can be considered a source that supplies the secondary chamber [40]. Silane diffuses inside the chamber, grafting along the glass surface at its bottom. Carefully, the secondary chamber was decoupled from the glass slide to ensure stability of the silane-deposited region. The distribution of grafted particles is measured to conclude the experiments. After diffusion, the slides are removed, separated from their secondary chamber, and placed in a foil-covered container to await staining.

Solutions of 5 µg/mL of Alexa Fluor 594 NHS Ester (A20004; Thermo Fisher, Fremont, CA, USA) in 1X PBS are prepared in foil-covered tubes (A37572; Thermo Fisher, Fremont, CA, USA). Underneath the foil, each tube is covered with polyimide tape (8997; 3M, St. Paul, MN, USA) to prevent additional light from interacting with the fluorescent solution. Once prepared, 200 µL of this solution is applied to the region of the silanized glass slides. These are incubated at 25 ${}^{\circ }C$ for 45 min, covered from light (Figure 1d). After staining, excess solution is removed and the slide is gently washed with 70% ethanol to remove any remaining unbonded fluorescent dye.

After staining and cleaning, the amine-conjugated slides are imaged within 24 h to avoid significant hydrolysis of the silane or photobleaching of the fluorophore. The slides are then placed on the stage of an Olympus IX83 Research Inverted Microscope and imaged with a 4X air objective at constant laser power and voltage (Figure 1e). The slides are imaged at 5 mm increments, from the opening of the secondary chamber (at x=0) to the dead-end of the chamber (at x=L). To control for variability, five images randomly selected along the width are collected at each *x*-location, and five randomly selected points within each of these images are measured for average fluorescence intensity using the software ImageJ. Mean intensity measurements thus serve as a direct proxy for the molecular surface coverage per unit area or, equivalently, the local fractional surface coverage of the silane monolayer. Each of these experimental trials is conducted at least three times to account for sample-to-sample variability. We found no statistically significant difference between the intensities measured at the secondary chamber opening (x=0) when the internal geometry of the secondary chamber was varied. As time passes during the diffusion process, eventually silane grafting at the base of the secondary chamber reaches a uniformly saturated state. However, prior to saturation, a gradient of silane is observed along the secondary chamber floor, moving from high concentrations of silane near the inlet to lower concentrations near the chamber dead-end. If the equivalent experiment is conducted without the secondary chamber, we observe no detectable gradient in our system.

### 2.2. Mathematical Model and Numerical Simulations

To rationalize our experimental observations, we propose a simple diffusion-grafting model which includes the passive diffusion of the vaporized silane into the chamber and molecular grafting onto the glass slide. The timescale associated with passive diffusion alone does not explain the deposited silane gradient observed in the experimental data. The diffusion timescale, $\tau_{x}$, is the characteristic timescale for a particle subject only to molecular diffusion to reach the dead-end of the secondary chamber. For our experimental parameters, this timescale is estimated to be on the order of $\tau_{x}=L^{2}/\kappa ∼$ 10 min, where $\kappa ∼0.1\;{\mathrm{mm}}^{2}/s$ is the molecular diffusion coefficient of gaseous silane estimated from prior experiments [35,41]. This timescale estimate is consistent with measurements from prior work where molecular diffusion alone was shown to be principally responsible for the gradient formation [41]. In contrast, in the absence of grafting, the gaseous silane density would quickly equilibrate in the interior secondary chamber in our system, long before the times where silane gradients are still observed in the experiments (on the order of hours). Introducing the grafting mechanism in the model disrupts the diffusion process by depleting silane particles at a high enough rate that a deposited gradient is formed and the secondary chamber becomes saturated only on extremely long timescales, long past diffusion time, as observed in the experiments.

**Figure 2:**
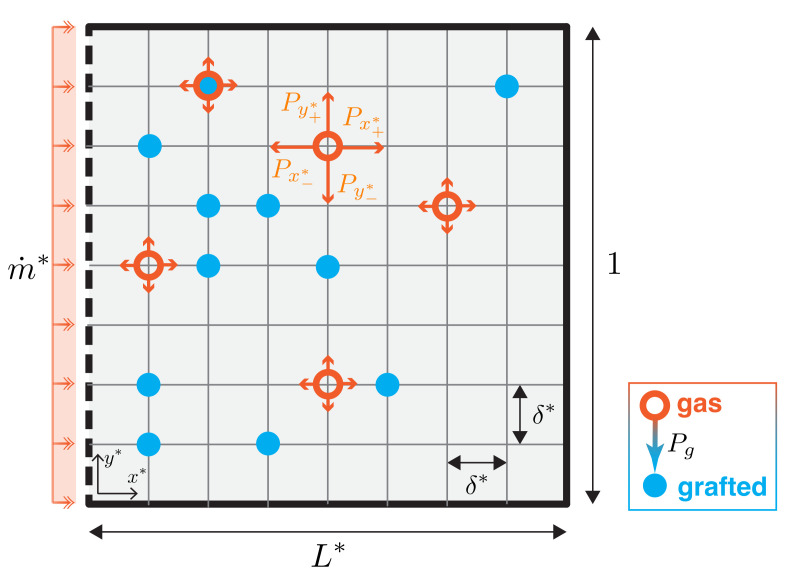
Schematic of 2D domain for Monte Carlo simulation. Particles enter uniformly at $x^{*}=0$ with flux ${\dot{m}}^{*}$ and undertake a symmetric random walk on a 2D lattice with step-size $\Delta x^{*}=\Delta y^{*}=\delta^{*}$ and probability $P_{x_{+}^{*}}=P_{x_{-}^{*}}=P_{y_{+}^{*}}=P_{y_{-}^{*}}=1/4$. At each time step, the particles have a probability $P_{g}$ of irreversibly grafting in place. Only one particle is allowed to graft at each grid site.

The present diffusion-grafting model is inspired by the work of Bautista et al. [42]. We use a Monte Carlo method to simulate the dynamics of individual gaseous silane particles undergoing passive diffusion; see Supplementary Materials for the full code implemented in MATLAB. The system is assumed to be in the dilute limit where particle-particle interactions can be neglected. As the particles diffuse within the secondary chamber, they have a finite probability of grafting on the glass slide beneath, at which point their position is fixed for the remainder of the simulation. In this work, we assume the grafting process is irreversible. Thus, we ultimately distinguish between two particle states: gas and grafted, as visualized in Figure 2 in orange and blue, respectively. Despite its relative simplicity, this model captures the principal physics of our system and reproduces the qualitative trends observed in the experiment.

For an initial APTES volume of 100 µL, and assuming all molecules are vaporized, we estimate an average molecular spacing of approximately 20 nm in the exterior primary chamber of volume $\approx 1.6\times 10^{7}$${\mathrm{mm}}^{3}$. For comparison, the average molecular spacing in a silane monolayer has been measured to be in the order of 1 nm, or less [43,44]. Hence, the density of the vaporous silane molecules available for grafting is relatively dilute in comparison to the density of molecules within a saturated silane monolayer.

We model the secondary chamber (a three-dimensional (3D) rectangular prism, Figure 1f) as a two-dimensional rectangular domain in the xy-plane with length *L* and width *w* (Figure 2). In our experiments the height *h* of the chamber is much smaller than its lateral dimensions (h≪w,L) so any vertical (*z*) concentration gradients equilibrate rapidly, justifying this simplification from the full 3D problem. We non-dimensionalize the spatial and time scales with respect to the width of the secondary chamber *w* which is maintained constant throughout the experimental investigations. The non-dimensional coordinates are defined as follows:

(1) $$x^{*}=\frac{x}{w},\;y^{*}=\frac{y}{w},\;z^{*}=\frac{z}{w},\;t^{*}=\frac{t}{\tau_{y}}=\frac{t}{w^{2}/\kappa },$$

where $\tau_{y}=w^{2}/\kappa$ is the diffusion time-scale in the transverse *y*-direction.

We assume there is a constant flux of vaporized silane diffusing into the chamber through its opening ($x^{*}=0$) towards its dead-end ($x^{*}=L^{*}$), which is rationalized in what follows. Fick’s first law defines mass flux, $\dot{m}$ (with [$\dot{m}$] = particles/s), as proportional to the concentration gradient across the area, *A*:

(2) $$\dot{m}\propto A\;\left(C_{o}-C_{i}\right).$$

In our case, the concentration gradient $C_{o}-C_{i}$ is through the opening area of the secondary chamber, A=w·h. The concentration of silane in the interior of the chamber ($C_{i}$) is assumed to remain relatively low due to the preeminence of grafting, that is, $C_{i}\ll C_{o}$. Furthermore, the volume of the primary chamber is much greater than that of the secondary diffusion chamber, and thus we can assume that the concentration outside the secondary chamber ($C_{o}$) remains approximately constant throughout the duration of the experiments. These considerations justify our assumption of constant flux of silane diffusing into the chamber, which greatly simplifies the model (as the diffusion mechanics in the *primary* diffusion chamber need not be simulated). We impose a constant flux of particles at the inlet, releasing particles uniformly distributed along the $y^{*}$-direction at $x^{*}=0$, at a fixed introduction rate ${\dot{m}}^{*}$ with [${\dot{m}}^{*}$] = particles/$\tau_{y}^{*}$.

The computational domain is spanned by a 2D grid with equal spacing in both directions, $\Delta x^{*}=\Delta y^{*}=\delta^{*}$. Particles undertake a symmetric random walk on the 2D lattice [45]; that is, at each time interval $\Delta t^{*}$, particles take a step of length $\delta^{*}$ in either the positive $x^{*}$-direction, the negative $x^{*}$-direction, the positive $y^{*}$-direction, or the negative $y^{*}$-direction with equal probability $P_{x_{+}^{*}}=P_{x_{-}^{*}}=P_{y_{+}^{*}}=P_{y_{-}^{*}}=1/4$. Each step is independent of the others [45].

Since diffusion timescales are defined as $\tau =\ell^{2}/\kappa$, where *ℓ* is the spatial dimension of interest and κ is the molecular diffusion coefficient, the diffusion time for silane spreading along the length of the chamber is equal to $L^{2}/\kappa$, while it is equal to $h^{2}/\kappa$ along the height of the chamber. As mentioned, in our domains L∼w, h≪w,L, and κ∼0.1${\mathrm{mm}}^{2}/s$ (consistent with [35,41]). For a symmetric random walk on a 2D lattice of step-size $\delta^{*}$, and as $\delta^{*}\to 0$ and $\Delta t^{*}\to 0$, the effective non-dimensional molecular diffusion coefficient $\kappa^{*}$, is known to be [45]:

(3) $$\kappa^{*}=\frac{{\left(\delta^{*}\right)}^{2}}{4\;\Delta t^{*}}.$$

At each time step, each particle can either diffuse to a different grid location by taking a single step $\delta^{*}$, as described above, or graft in place with probability $P_{g}$. As soon as grafting occurs, the particle stops moving for the remainder of the simulation. Only one particle can graft at each site in order to model the silane monolayer [46], while multiple gas particles can co-exist at the same grid-point, simulating the finite height of the secondary chamber. Our simulations are run to times far past the longitudinal diffusion time, as is the case in the experiments. The boundary conditions at the walls are billiard-like, with particles being reflected back into the domain if they are computed to cross a boundary. Although this is a slow method with a convergence rate that scales with the number of particles *n* as $1/\sqrt{n}$, this approach is well-suited to simulate our discrete probabilistic system [42,45,47]. We compare experimental data measured between 4 and 24 h with 4-h increments, with simulation results at six times taken at a fixed increment, $t_{i}^{*}$. We compute ensemble averages by carrying out *r* runs with the same parameters. For all simulation results reported herein, we take r=500.

Our reference case is chosen to be an experiment with an interior secondary chamber of dimensions L=30 mm, w=30 mm, and h=1 mm; here, we select δ=0.3 mm and $\Delta t=2.25\times 10^{-1}$ s such that the dimensional diffusion coefficient is $\kappa =0.1\;{\mathrm{mm}}^{2}/s$. We model this geometry as a 2D domain in the non-dimensional Monte Carlo simulation with $L^{*}\times w^{*}=1\times 1$ (Figure 2). For $\delta^{*}=0.01$ and $\Delta t^{*}=2.5\times 10^{-5}$, the non-dimensional molecular diffusion coefficient is $\kappa^{*}=1$. Hence, diffusion time in the longitudinal $x^{*}$-direction, $\tau_{x}^{*}=1$, is reached after $4\times 10^{4}$ time-steps, corresponding to $\tau_{x}^{*}{\dot{m}}^{*}=1000$ particles being released. We use our reference domain to determine a suitable set of free parameters of the model, as discussed in the following section.

We then extend our results to consider rectangular prism secondary chambers of other internal dimensions. First, we keep the chamber width fixed at 30 mm ($w^{*}=1$) and its height fixed at 1 mm ($h^{*}=0.03$), and vary its length from 30 mm to 20 mm and 50 mm ($L^{*}=1$, $L^{*}=0.66$, and $L^{*}=1.66$, respectively). Then, we keep the chamber width and length both fixed at 30 mm ($w^{*}=L^{*}=1$) and vary its height from 1 mm to 2 mm and 4 mm ($h^{*}=0.03$, 0.06, and 0.13, respectively). We simulate changes in the chamber height by increasing the flux of particles at the inlet proportionally to the chamber opening cross-sectional area $A^{*}=w^{*}\;\cdot \;h^{*}$, in accordance with Equation (2). Furthermore, we anticipate that the probability of grafting $P_{g}$ at each time step will be reduced for increased $h^{*}$, as there is a reduced chance that the molecule is in proximity of the grafting substrate at a given instance in time. Since the time for a particle to traverse the box height will scale with the diffusive transport time in the vertical $z^{*}$-direction, we assume an inverse quadratic relationship between $P_{g}$ and $h^{*}$, that is, $P_{g}=\alpha /{\left(h^{*}\right)}^{2}$.

Table 1 summarizes the parameters explored by our Monte Carlo simulations, which are compared directly to experimental measurements in the following section.

## 3. Results

**Figure 3:**
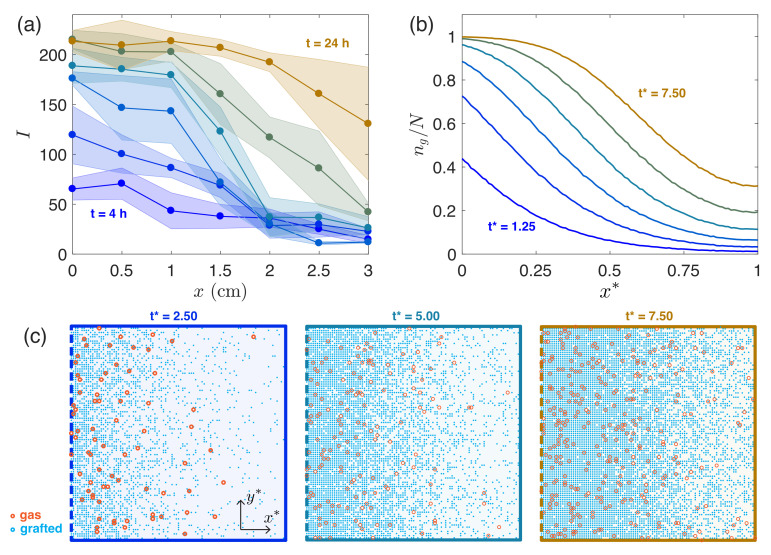
(**a**) Experimental measurements of fluorescent intensity profile at 6 different times, separated by 4 h. Interior secondary chamber dimensions: L×w×h=30×30×1 mm. Five images are taken at 5 mm increments and five randomly selected points within each of these images is measured for average fluorescence intensity. Shaded regions on each side represent one standard deviation of measurements obtained from at least three independent experiments. (**b**) Prediction of model at six evenly spaced times (for $t^{*}>\tau_{x}^{*}$) with $P_{g}=5\times 10^{-4}$, $L^{*}=1$, $w^{*}=1$, $h^{*}=0.03$, ${\dot{m}}^{*}=1000$. Each curve for $t^{*}=$ 1.25, 2.50, 3.75, 5.00, 6.25, and 7.50, represents the average result over 500 runs. (**c**) Views of vaporized (orange) and grafted (blue) silane particle locations within a single simulation for times $t^{*}=$ 2.50, 5.00, and 7.50 (left to right).

**Figure 4:**
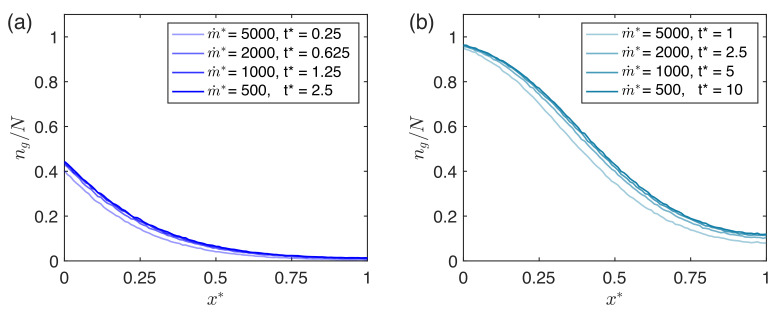
Dependence of Monte Carlo prediction on choice of ${\dot{m}}^{*}$ and $t_{i}^{*}$. Note that in each panel, the number of particles that have entered the system (at the time of observation) is held fixed, i.e., the product ${\dot{m}}^{*}t^{*}$ is a constant. (**a**) ${\dot{m}}^{*}t^{*}=1250$ particles. (**b**) ${\dot{m}}^{*}t^{*}=5000$ particles.

**Figure 5:**
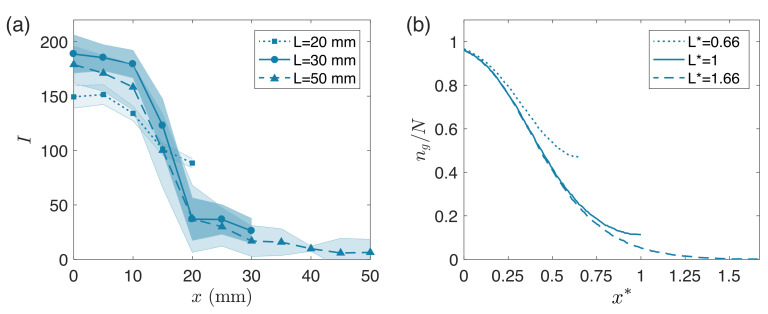
(**a**) Experimental measurements of fluorescent intensity profile at 16 h for three different secondary interior chamber lengths. Interior secondary chamber dimensions: L×w×h=L×30mm×1mm, with L= 20 mm, 30 mm, or 50 mm. Shaded regions on each side represent one standard deviation of measurements obtained from at least three independent experiments. (**b**) Prediction of model at $t^{*}=5.00$ for three different domain lengths with $P_{g}=5\times 10^{-4}$, $w^{*}=1$, $h^{*}=0.03$, ${\dot{m}}^{*}=1000$, and $L^{*}=0.66$, $L^{*}=1$, or $L^{*}=1.66$. Each curve represents the average result over 500 runs.

**Figure 6:**
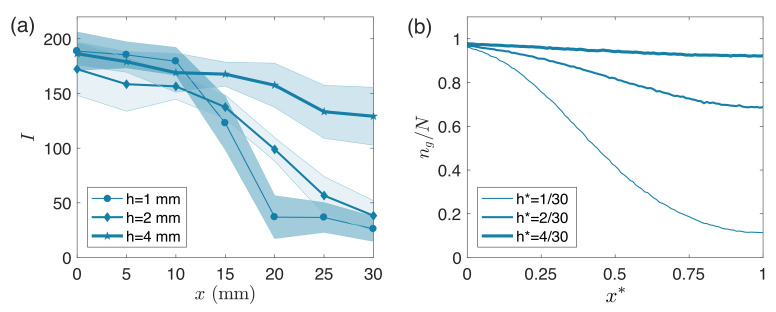
(**a**) Experimental measurements of fluorescent intensity profile at 16 h for three different secondary interior chamber heights. Interior secondary chamber dimensions: L×w×h=30mm×30mm×h, with h= 1 mm, 2 mm, or 4 mm. Shaded regions on each side represent one standard deviation of measurements obtained from at least three independent experiments. (**b**) Prediction of model at $t^{*}=5.00$ for three different domain heights with $w^{*}=$ 1 and $L^{*}=$ 1. To simulate height $h^{*}=$ 0.03, we use $P_{g}=5\times 10^{-4}$ and ${\dot{m}}^{*}=1000$; for $h^{*}=$ 0.06, we use $P_{g}=5/4\times 10^{-4}$ and ${\dot{m}}^{*}=2000$; and for $h^{*}=$ 0.13, we use $P_{g}=5/16\times 10^{-4}$ and ${\dot{m}}^{*}=4000$. Each curve represents the average result over 500 runs.

We report our experimental and Monte Carlo simulation results grouped by changes in the secondary interior chamber geometry. We compare the simulation results to the experimental reference case (Figure 3) in order to define the parameters used across all further simulations, and further justify our choice of parameters in Figure 4. In Figure 5 and Figure 6 we show how changing the interior secondary chamber dimensions affects the deposited silane gradient. In Figure 3, Figure 5 and Figure 6, the (a) panels show experimental results with shaded regions on each side representing one standard deviation of measurements obtained from at least three independent experiments. All (b) panels show Monte Carlo simulation results averaged across r=500 runs.

Figure 3 shows experimental and simulation results for our reference case (Monte Carlo parameters reported in shaded row of Table 1). In Figure 3a we report experimental measurements of fluorescent intensity profiles due to a deposited silane gradient. The six curves show measurements 4 h apart (t = 4, 8, 12, 16, and 24 h). A decrease in intensity (corresponding to less silane grafting) is reported across the experimental timescale as *x* increases, confirming the presence of a deposited silane gradient and further indicating that the amount of silane deposited has not saturated the available binding sites on the glass slide at the times presented. In general, the overall fluorescence intensity at any point in space increases steadily as time progresses in the experiment.

In Figure 3b we report simulation results with parameters: $P_{g}=5\times 10^{-4}$, $L^{*}=1$, $w^{*}=1$, $h^{*}=0.03$, and ${\dot{m}}^{*}=1000$. The six times shown are past the diffusion time scale ($t^{*}>\tau_{x}^{*}$) and are integer multiples of the fitted time increment $t_{i}^{*}=$ 1.25, that is, $t^{*}=$ 1.25, 2.50, 3.75, 5.00, 6.25, and 7.50. We plot the ratio of grafted particles to the number of binding sites at a given $x^{*}$-location, $n_{g}\left(x^{*}\right)/N$, *vs* the simulated domain length ($x^{*}$) over time. We note similar trends to the experimental measurements, highlighting the increase in the number of grafted particles as time passes, and the decrease when penetrating deeper into the chamber domain.

We utilize this chamber cross-section as our reference case and use it to fix the free simulation parameters: $P_{g}$, ${\dot{m}}^{*}$, and $t_{i}^{*}$. For higher grafting probabilities, the gradients become less steep and the simulated chamber saturates faster, as the binding sites are swiftly occupied. We fix the introduction rate such that the relevant observation times are beyond the diffusion timescale ($\tau_{x}^{*}=1$). As ${\dot{m}}^{*}$ decreases further, nearly identical quantitative grafted particle gradients are observed in simulation, albeit on even longer timescales. More precisely, if the product of ${\dot{m}}^{*}t^{*}$ (which corresponds to the number of particles introduced prior to the observation time $t^{*}$) is fixed, the quantitative prediction for the grafted particle distribution converges to a single curve, assuming that ${\dot{m}}^{*}$ is selected to be sufficiently small. This feature of the model is demonstrated for the reference case in Figure 4. For our simulations, we thus opt to select a ${\dot{m}}^{*}$ value that is sufficiently small to ensure convergence, without being too small that the total simulation time becomes unwieldy. Thus in practice, the product ${\dot{m}}^{*}t_{i}^{*}$ represents a single fitting parameter (as opposed to fitting ${\dot{m}}^{*}$ and $t_{i}^{*}$ independently).

Figure 3c shows three snapshots of the two-state system in our Monte Carlo simulations: particles are either grafted (blue) or vaporized (orange). The number of grafted particles increases from $t^{*}=$ 2.50 (left) to $t^{*}=$ 5.00 (middle) and to $t^{*}=$ 7.50 (right) and accumulates towards the domain opening (left, dashed boundary).

Figure 5 compares experimental and simulation results across secondary interior chambers of three lengths: 20 mm, 30 mm, and 50 mm (Monte Carlo parameters reported in top three rows of Table 1). In Figure 5a, we report experimental measurements of the fluorescent intensity profile due to the deposited gradient from 100 µL of vaporized silane. Three curves (each corresponding to a different chamber length) are superimposed at a single time (t= 16 h). We note that, near the inlet, similar gradient profiles are observed independent of chamber length. For the two longer chambers, a steady decrease in fluorescence intensity is observed for x>20 mm, while for the shortest chamber (L= 20 mm), the same fluorescence decrease is not measured as *x* approaches the chamber dead-end. In Figure 5b, we report simulation results with parameters: $P_{g}=5\times 10^{-4}$, $h^{*}=0.03$, and ${\dot{m}}^{*}$ = 1000. We use $L^{*}=0.66$, $L^{*}=1$, and $L^{*}=1.66$ to simulate the 20 mm, 30 mm, and 50 mm chamber lengths, respectively. The only time shown ($t^{*}=$ 5.00) is long past diffusion time ($t^{*}\gg \tau_{x}^{*}$) and corresponds to 16 h, that is, $t^{*}=4t_{i}^{*}$. We plot the particle ratio, $n_{g}\left(x^{*}\right)/N$, *vs* the simulated domain width ($x^{*}$) for each of the three chambers. We note similar trends to the experimental measurements, highlighting comparable gradient profiles near the inlet regardless of the chamber length. Additionally, simulations capture the difference in grafting as silane penetrates further into the chamber lengths, distinguishing behaviors between the two longer chambers and the shortest one (with $L^{*}=0.66$).

Finally, Figure 6 compares experimental and simulation results across secondary interior chambers of three heights: 1 mm, 2 mm, and 4 mm (Monte Carlo parameters reported in the shaded and bottom two rows of Table 1). In Figure 6a, three fluorescent intensity curves (each corresponding to a different chamber height) are superimposed at a single time (t= 16 h). For all of the secondary chamber heights, we report a statistically significant difference in fluorescence between the inlet at x=0 mm and the dead-ends at x=30 mm. Experimental data show how fluorescence values at the inlet are independent of secondary chamber height, while fluorescence values at the dead-ends are not. Recorded fluorescence at x= 30 mm is significantly higher for the 4 mm chamber height compared to the two shortest chambers. In Figure 6b we report simulation results with parameters $L^{*}=$ 1 and $w^{*}=$ 1. We use $h^{*}=$ 0.03, $h^{*}=$ 0.06, and $h^{*}=$ 0.13 to simulate the 1 mm, 2 mm, and 4 mm chamber heights, respectively. While the $h^{*}=$ 0.03 chamber corresponds to our reference case with parameters reported in the shaded row of Table 1, to simulate the $h^{*}=$ 0.06 case we use $P_{g}=5/4\times 10^{-4}$ and ${\dot{m}}^{*}=2000$, and for the $h^{*}=$ 0.13 case we use $P_{g}=5/16\times 10^{-4}$ and ${\dot{m}}^{*}=4000$. We plot the particle ratio, $n_{g}\left(x^{*}\right)/N$, *vs* the simulated domain length ( $x^{*}$) for each of the three chambers at $t^{*}=$ 5.00. Although the curves are not quite as aligned to the experimental results as in Figure 3 and Figure 5, we can still note similar overall trends between panels (a) and (b). Simulations agree with experiments in showing how the number of grafted particles near the inlet is independent of secondary chamber height, while this is not true at the dead-end. Specifically, we highlight how increasing the height of the secondary chamber directly reduces the steepness of the gradient at the same observation time. Finally, simulations capture the significantly higher grafting recorded for the $h^{*}=0.13$ (h=4 mm) chamber height when the glass-slide sites seem to be nearly saturated.

We can capture the trends predicted by the numerical model by a simple physically-motivated scaling analysis, as follows. A simulated particle that enters the secondary chamber must wait a typical delay time $t_{g}^{*}=\Delta t^{*}/P_{g}=\Delta t^{*}{\left(h^{*}\right)}^{2}/\alpha$ before grafting in place. As discussed previously, the diffusive timescale for a vaporized particle to traverse the length of the chamber is $\tau_{x}^{*}={\left(L^{*}\right)}^{2}/\kappa^{*}$ or $\tau_{x}^{*}=4{\left(L^{*}\right)}^{2}\Delta t^{*}/{\left(\delta^{*}\right)}^{2}$ (via Equation (3)). Thus, if $t_{g}^{*}\gg \tau_{x}^{*}$, the particle will have time to fully explore the chamber length before grafting, ultimately eliminating any gradient resulting from the spatial asymmetry of the particle introduction. In the opposite limit $t_{g}^{*}\ll \tau_{x}^{*}$, the particles will rapidly graft near the inlet of the secondary chamber or shortly after they arrive at an unoccupied grafting site. This limit ultimately yields a very steep gradient. We can thus define a non-dimensional parameter *G* that compares these two times scales and provides a sense of the steepness of the gradient (relative to the box length $L^{*}$) as a function of the parameters of the model:

(4) $$\frac{\tau_{x}^{*}}{t_{g}^{*}}∼\frac{P_{g}{\left(L^{*}\right)}^{2}}{{\left(\delta^{*}\right)}^{2}}=\frac{\alpha {\left(L^{*}\right)}^{2}}{{\left(h^{*}\right)}^{2}{\left(\delta^{*}\right)}^{2}}=G.$$

**Figure 7:**
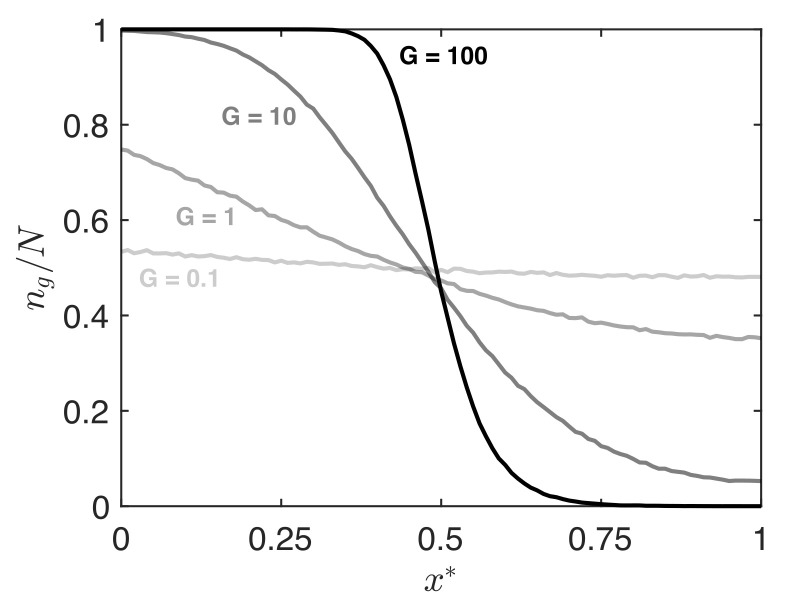
Comparison of predicted surface coverage profiles as a function of parameter *G*. Each curve is extracted from Monte Carlo simulations at the time when the average surface coverage reaches Φ=0.50. In all cases shown, $\delta^{*}=0.01$ and $L^{*}=1$, with the value of $P_{g}$ varied to obtain the different *G* factors (Equation (4)). Each curve represents the average result over 500 runs.

G≪1 corresponds to a gentle gradient and G≫1 corresponds to a steep gradient. It is important to note that within a given simulation, the overall gradient steepness is not constant, but rather varies as a function of time (e.g., Figure 3). For instance, if one waits long enough, all gradients will vanish regardless of the *G* factor. Thus the *G* factor is useful in comparing simulations at intermediate times, where the average fractional surface coverage Φ is comparable between cases. In Figure 7, we compare the predicted surface gradients for different values of *G*, for observation times at which Φ=0.50. The steepness of the gradient correlates directly with the parameter *G*. When *G* is of order 1–10, we predict a gradient that smoothly spans the length $L^{*}$ of the secondary chamber, as in the cases we feature in the present work (see Table 1).

## 4. Discussion

In this manuscript, we have shown how a controllable gradient of surface properties can be constructed using vapor-phase silanization. Experimentally, the gradient of grafted silane is accomplished using a primary diffusion chamber to contain vaporized silane in vacuum, and a single-opening secondary diffusion chamber attached to the top of a substrate. In all cases presented, it is observed that more silane grafts near the opening of the secondary chamber leading to quantifiable gradient formation of surface properties. Additionally, longer experimental times correlate to more overall silane deposition. Modeling efforts employing Monte Carlo simulations capture the physical behavior of deposited silane and can be used to guide *ad hoc* gradient design in silanization processes. The simple diffusion-grafting model presented is sufficient to capture the key physics and thus the primary trends observed in the experiment. In particular, we note that increasing the length of the secondary chamber does not appreciably affect the gradient (Figure 5); although, if re-scaled by the chamber length *L*, the gradient becomes relatively steeper for increasing length. Augmenting the secondary chamber height *h* is shown to reduce the gradient steepness (Figure 6).

While the primary trends observed in the experiment are well captured by our 2D diffusion-grafting model, not all details can be predicted with such a simple model. For example, we are utilizing a 2D domain to simulate a 3D system. This choice may have more significant effects when trying to account for variations in the chamber height explored in the experiment; in fact, results reported in Figure 6 are where the experiment-to-simulation agreement seems to be less quantitative. Extending this model to a full 3D Monte Carlo method could improve the comparison with experimental observations. A further limitation of our diffusion-grafting model is that it relies on an experimental observation to define its three free parameters ($P_{g}$, ${\dot{m}}^{*}$, and $t_{i}^{*}$); in the present work, data from the reference experimental case of a 30×30×1 mm (1×1×0.03 in non-dimensional coordinates) interior secondary chamber is leveraged to define default parameter values for our Monte Carlo method. A more elaborate grafting mechanism that takes into account the occupancy of a particle’s neighboring sites may make this model more accurate [42]. The probability of grafting could be higher for a particle joining a cluster of occupied grafting sites. Such a mechanism may make it simpler to extend the use of this model to describe deposition of other compounds.

In the experiment, there are a number of future directions that could help generalize the present results and further enhance the passive control mechanism identified in this work. In particular, different vaporized volumes of silane could be tested incrementally, to assess the effect of primary chamber concentration on the timescales and gradient shape. Furthermore, other gaseous compounds and substrates should be tested to broaden the possible applications of the technique. Finally, different shapes of the secondary diffusion chamber could also be tested in the future (e.g., cylindrical, trapezoidal, triangular, chambers with increasing or decreasing height, etc.), which would potentially allow for arbitrary 2D gradient profiles to be observed and applied in biotechnology.

In related work, experiments have demonstrated that utilizing such gradients in conjunction with liquid lithography can create biomedical micropillar stamps with controlled microtip curvature to be used for in vitro crypts with measurable effects on cell behavior [22]. The technique presented in this work could increase the microstructural relevance of a lab-on-a-chip intestinal crypt model for use in tissue engineering, regenerative medicine, and drug screening, and significantly improve the effectiveness of this culture method in mimicking in vivo intestinal behavior.

While our immediate motivation for developing the technique presented is for the liquid lithography process in organ-on-a-chip applications, we anticipate that our passive technique will be useful for a much broader range of physical systems where a wettability gradient may also prove useful [34], such as in microfluidics [48], studies of cell attachment and growth [49], or even for fog collection [50]. 

## Figures and Tables

**Table 1:** Secondary chamber dimensions and parameters for Monte Carlo simulations (and corresponding experimental dimensions). The shaded, second row reports the reference case dimensions and parameters. The top rows show the dimensions and parameters for chambers of different length $L^{*}$, (first row) with $L^{*}=0.66$ in the first row and (third row) $L^{*}=1.66$ in the third row. The bottom rows show the dimensions and parameters for chambers with increasing heights $h^{*}$, with $h^{*}=0.06$ in the penultimate row and $h^{*}=0.13$ in the last row.

	$w^{*}\;\left(w\right)$	$L^{*}\;\left(L\right)$	$h^{*}\;\left(h\right)$	${\dot{m}}^{*}$	$P_{g}$	*G* [Equation (4)]
	1(30mm)	0.66(20mm)	0.03(1mm)	1000	$5\times 10^{-4}$	2.18
Reference case	1(30mm)	1(30mm)	0.03(1mm)	1000	$5\times 10^{-4}$	5.00
	1(30mm)	1.66(50mm)	0.03(1mm)	1000	$5\times 10^{-4}$	13.8
	1(30mm)	1(30mm)	0.06(2mm)	2000	$5/4\times 10^{-4}$	1.25
	1(30mm)	1(30mm)	0.13(4mm)	4000	$5/16\times 10^{-4}$	0.31

## Data Availability

The data presented in this study are available for review upon request; please reach out to rhoward1@live.unc.edu.

## Supplementary Materials

The Monte Carlo code developed for this manuscript is available online at https://www.mdpi.com/article/10.3390/mi12111360/s1.
