# Supplementary material for: Passive Control of Silane Diffusion for Gradient Application of Surface Properties
Source: Micromachines (Basel). 2021 Nov 4;12(11):1360. doi: 10.3390/mi12111360 (PMC8620173; doi:10.3390/mi12111360)
Supplement: Supplementary file 1 [file micromachines-12-01360-s001.zip › micromachines-1369345-supplementary.pdf]

Supplementary Material for *Passive control of silane diffusion for gradient application of surface properties*

Monte Carlo Code

Riley L. Howard, Francesca Bernardi, Matthew Leff, Emma Abele, Nancy L. Allbritton and Daniel M. Harris

This document contains six MATLAB functions (one main function, and five helper functions) that can be used to run Monte Carlo simulations of silane diffusion-grafting model. The first function (MC_AVG) calls the other five. To run, use the following format in the command line:

```
MC_AVG(xr,ng,pgraft,steps,releasetime,N,tfinal,numsim)
```

Inputs: *xr* = x-coordinate of simulated right boundary,

ng = number of gridpoints in the x-direction,

pgraft = probability of grafting,

tfinal = simulations end-time,

steps = number of timesteps between 0 and tfinal,

1 particle is released after *releasetime* steps,

N = number of available particles,

numsim = number of independent simulations run who outputs will be averaged.

Output: The code outputs two files in the main directory where all the codes are saved:

H.mat = averaged histogram matrix for the number of grafted particles at each of the six snapshots in time.

binc.mat = location of the bin centers for the histogram matrix saved.

1) Main function: MC_AVG.

```
function MC_AVG(xr,ng,pgraft,steps,releasetime,N,tfinal,numsim)

ntimes = 6; % Number of snapshots in time between 0 and tfinal

% Define the simulated chamber domain: [xl,xr] x [yb,yt]
xl = 0; % xr is an input
yb = 0; yt = 1;
```

```
h = 0.01; % step-size in the x-direction

% Initialize histogram matrix
Hsum = zeros(ntimes,ng+1);

% Run the same simulation numsim times and average across the results
for i = 1:numsim
    % Monte Carlo code running one simulation for diffusion-grafint model
    [X,Y,G,A,~] =
MC_2D_LL_Step3b_flux_AVG(xl,xr,yb,yt,pgraft,steps,releasetime,N,tfinal);
    % Storing the x- and y-coordinates of gas-phase and grafted particles in separate
    % histogram matrices
    [Xgr,~,~,~] = grafted_gas_flux_save(ntimes,N,X,Y,G,A);
    % Saving the binning center locations for the histogram matrices
    [H,binc] = binning_centers_save(ntimes,xl,xr,h,ng,Xgr);
    % Storing histogram matrices
    Hsum = Hsum + H;
end

% Compute average across numsim simulations
Havg = Hsum./numsim;

% Saving the averaged histogram matrix and bin center matrix
save('H.mat','Havg');
save('binc.mat','binc');

end
```

i) Helper function 1: MC_2D_LL_Step3b_flux_AVG.

```
function [X,Y,G,A,T] =
MC_2D_LL_Step3b_flux_AVG(xl,xr,yb,yt,pgraft,steps,releasetime,N,tfinal)

format short g

dig = 6; % Digits of accuracy
dt = round(tfinal/steps,dig); % Time-step
ngy = 100; % Number of gridpoints in the y-direction between yb and yt
h = round(1/ngy,dig); % Step-size in the y-direction

% Initialize state vectors
grafted = zeros(1,N); % Two states: grafted = 0 is for gas-phase, grafted = 1 is for
grafted particles
```

```
 active = zeros(1,N); % Two states: active = 0 is a latent particle, active = 1 is for
particles introduced in the chamber
 active(1) = 1; % At t = 0 one particle is introduced in the simulation chamber
 release = 2; % After "releasetime" time-steps, one more particle is released

 % Set up initial condition at t = 0
 x = zeros(1,N); % On the left-end of the domain
 % Randomly distributing particles along the y-axis allowing them to take only discrete
locations along the grid
 y = round(floor((ngy+1)*rand(1,N))/ngy,dig);
 % Initializing vectors to store variables at six intermediate times
 X = zeros(6,N);
 Y = zeros(6,N);
 G = zeros(6,N);
 A = zeros(6,N);
 T = zeros(6,1);

 % Time
 t = zeros(1,steps+1); % time vector
 % Number of time steps at six intermediate times
 steps1 = round(steps/6,dig); steps2 = steps1*2; steps3 = steps1*3;
 steps4 = steps1*4; steps5 = steps1*5;

 % Monte Carlo simulation
 for i = 1:steps % Time stepping
     for j = 1:N % Looping over each particle
         if active(j) == 1
             % If particle j is not grafted
             if grafted(j) == 0
                 % "Coin flip" to pick whether particle grafts or moves
                 coinG = rand(1);
                 if coinG <= pgraft % The particle should graft if the site is empty
                     % Check if there is already a grafted particle with
                     % coordinates (x(j),y(j))
                     diff = 0;
                     for k = 1:N
                         if grafted(k) == 1
                             grcheckx = round(x(k) - x(j),dig);
                             grchecky = round(y(k) - y(j),dig);
                             % If there is already a grafted particle with coordinates
(x(j),y(j))
                             if grcheckx == 0 && grchecky == 0
                                 % then diffuse
                                 [x(j),y(j)] = coin4(x(j),y(j),h,dig);
                                 diff = 1;
                                 break
```

```
                        end
                    end
                end
                % If there is no grafted particle at (x(j),y(j))
                if diff == 0
                    % then graft
                    grafted(j) = 1;
                end
            else % Otherwise, the particle diffuses
                [x(j),y(j)] = coin4(x(j),y(j),h,dig);
            end

            % Set up a rectangular domain (x,y) = [xl,xr] x [yb,yt] with
            % billiard-like reflections at the four walls
            % Bouncing particles back into the channel for y < yb and y > yt
            while y(j) > yt
                y(j) = round(y(j)-h,dig);
            end
            while y(j) < yb
                y(j) = round(y(j)+h,dig);
            end
            % Bouncing particles back into the channel for x < xl and x > xr
            while x(j) > xr
                x(j) = round(x(j)-h,dig);
            end
            while x(j) < xl
             x(j) = round(x(j)+h,dig);
            end
        end
    end
end

% Adding in one more particle after "releasetime" time-steps
if mod(i,releasetime) == 0
    active(release) = 1;
    release = release + 1;
end

% Saving the particle data at six instances in time
if i == steps1
    [X(1,:),Y(1,:),G(1,:),A(1,:),T(1)] =
save_matrices_flux(dt,i,dig,x,y,grafted,active);
elseif i == steps2
    [X(2,:),Y(2,:),G(2,:),A(2,:),T(2)] =
save_matrices_flux(dt,i,dig,x,y,grafted,active);
elseif i == steps3
```

```
        [X(3,:),Y(3,:),G(3,:),A(3,:),T(3)] =
save_matrices_flux(dt,i,dig,x,y,grafted,active);
    elseif i == steps4
        [X(4,:),Y(4,:),G(4,:),A(4,:),T(4)] =
save_matrices_flux(dt,i,dig,x,y,grafted,active);
    elseif i == steps5
        [X(5,:),Y(5,:),G(5,:),A(5,:),T(5)] =
save_matrices_flux(dt,i,dig,x,y,grafted,active);
    elseif i == steps
        [X(6,:),Y(6,:),G(6,:),A(6,:),T(6)] =
save_matrices_flux(dt,i,dig,x,y,grafted,active);
    end
    t(i+1) = round(dt*i,dig); % Update time vector
end

end
```

ii) Helper function 2: coin4.

```
function [xv,yv] = coin4(xv,yv,h,dig)

% "Coin flip" to pick direction of diffusion among 4 equally probable options: North,
South, East, West
coin = floor(4*rand(1));

% 0 = N, 1 = S, 2 = E, 3 = W
if coin == 0
    yv = round(yv+h,dig);
elseif coin == 1
    yv = round(yv-h,dig);
elseif coin == 2
    xv = round(xv+h,dig);
else
    xv = round(xv-h,dig);
end

end
```

iii) Helper function 3: save_matrices_flux.

```
function [X,Y,G,A,T] = save_matrices_flux(dt,i,dig,x,y,grafted,active)
```

```
X = x;
Y = y;
G = grafted;
A = active;
T = round(dt*(i-1),dig);

end
```

iv) Helper function 4: grafted_gas_flux_save.

```
function [xgr,ygr,xgas,ygas] = grafted_gas_flux_save(ntimes,N,x,y,grafted,active)

% Save x- and y- coordinates of grafted and gas-phase particles in four separate
matrices
countgraft = 1;
countgas = 1;

for i = 1:N
    if active(ntimes,i) == 1
        if grafted(ntimes,i) == 1
            xgrn(countgraft) = x(ntimes,i);
            ygrn(countgraft) = y(ntimes,i);
            countgraft = countgraft + 1;
        else
            xgasn(countgas) = x(ntimes,i);
            ygasn(countgas) = y(ntimes,i);
            countgas = countgas + 1;
        end
    end
end

lgr = length(xgrn);
lgas = length(xgasn);

% Define x- and y-ccordinate vectors for grafted and gas-phase particles
% including NaN for each element where there isn't a particle
xgr = zeros(ntimes,lgr); xgr(ntimes,:) = xgrn; xgr(1:ntimes-1,:) = NaN;
xgas = zeros(ntimes,lgas); xgas(ntimes,:) = xgasn; xgas(1:ntimes-1,:) = NaN;

ygr = zeros(ntimes,lgr); ygr(ntimes,:) = ygrn; ygr(1:ntimes-1,:) = NaN;
ygas = zeros(ntimes,lgas); ygas(ntimes,:) = ygasn; ygas(1:ntimes-1,:) = NaN;

for j = 1:ntimes-1
```

```
    countgraft = 1;
    countgas = 1;
    for i = 1:N
        if active(j,i) == 1
            if grafted(j,i) == 1
                xgr(j,countgraft) = x(j,i);
                ygr(j,countgraft) = y(j,i);
                countgraft = countgraft + 1;
            else
                xgas(j,countgas) = x(j,i);
                ygas(j,countgas) = y(j,i);
                countgas = countgas + 1;
            end
        end
    end
end

end
```

v) Helper function 5: binning_centers_save

```
function [H,binc] = binning_centers_save(ntimes,xl,xr,h,ng,xgr)

% Define edges for binning in the x-direction
xg = zeros(1,ng+2);
index = 1;

% Each bin contains one grid line in the y-direction: all particles with
% the same y-coordinate are binned together
for j=(xl-h/2):h:(xr+h/2)
    xg(index) = j;
    index = index + 1;
end

% Generate histogram without printing figure and save histogram values
for i = 1:ntimes
    xgrv = xgr(i,:);
    figure('visible','off')
    Hv = histogram(xgrv,xg);
    hv = Hv.Values;
    H(i,:) = hv;
end

% Save bin centers
```

```
binc = Hv.BinEdges(1:end-1) + Hv.BinWidth/2;

end
```

For questions reach out to corresponding author Francesca Bernardi at fbernardi@wpi.edu.
